# Hydraulic Integrated Interconnected Regenerative Suspension: Modeling and Characteristics Analysis

**DOI:** 10.3390/mi12070733

**Published:** 2021-06-22

**Authors:** Sijing Guo, Liang Chen, Xikai Wang, Junyi Zou, Sanbao Hu

**Affiliations:** 1 School of Automotive Engineering, Wuhan University of Technology, Wuhan 430070, China; sijingg@whut.edu.cn (S.G.); 262030@whut.edu.cn (L.C.); wxk704051999@foxmail.com (X.W.); 2 Hubei Key Laboratory of Advanced Technology for Automotive Components, Wuhan University of Technology, Wuhan 430070, China; 3 School of Automotive and Traffic Engineering, Wuhan University of Science and Technology, Wuhan 430070, China; zoujunyi@wust.edu.cn

**Keywords:** energy harvesting, hydraulic energy-harvesting shock absorber, hydraulic interconnected suspension, vibration characteristics, vehicle dynamics

## Abstract

A novel suspension system, the hydraulic integrated interconnected regenerative suspension (HIIRS), has been proposed recently. This paper demonstrates the vibration and energy harvesting characteristics of the HIIRS. The HIIRS model is established as a set of coupled, frequency-dependent equations with the hydraulic impedance method. The mechanical–fluid boundary condition in the double-acting cylinders is modelled as an external force on the mechanical system and a moving boundary on the fluid system. By integrating the HIIRS into a half car model, its free and forced vibration analyses are conducted and compared with an equivalent traditional off-road vehicle. Results show that the natural frequency and the damping ratio of the HIIRS-equipped vehicle are within a proper range of a normal off-road vehicle. The root mean square values of the bounce and roll acceleration of the HIIRS system are, respectively, 64.62 and 11.21% lower than that of a traditional suspension. The average energy harvesting power are 186.93, 417.40 and 655.90 W at the speeds of 36, 72 and 108 km/h for an off-road vehicle on a Class-C road. The results indicate that the HIIRS system can significantly enhance the vehicle dynamics and harvest the vibration energy simultaneously.

## 1. Introduction

Since the issue of energy depletion was raised, energy harvesting technology has received extensive attention [1,2,3]. In the automotive industry, extensive research and applications, including braking energy harvesting [4], exhaust thermo-electric recovery [5] and suspension vibration energy recovery [6,7,8], have also been conducted. Among them, braking energy recovery were gradually applied in electric vehicles. Considerable research has been carried out on energy harvesting suspensions. Zuo et al. [9] proved that the harvestable power in the suspensions of a middle-sized vehicle was 100–400 W, when driven at 60 mph on good and average roads. Zhang et al. [10] harvested 46 W from the suspension system of a passenger car. The research indicated that the energy harvesting suspension is feasible in theory and practice.

Among all traffic accidents, a large number of accidents are due to vehicle rollovers. It is reported that over one-third of 4WD fatalities involved rollover [11]. A percentage of 18.9 of rollover-related fatal crashes happened in the year of 2014 in the United States [12]. Suspension systems play a key role in reducing vehicle roll rates, and superior suspensions can greatly reduce the vehicular rolling propensity. Hydraulic interconnected suspension (HIS), with lower cost and easier structure than semi-active and active suspensions, was verified to be useful in reducing the roll rate [13] and, thus, became a potential alternative to the transmission of conventional suspensions.

To simultaneously enhance the vehicle safety and harvest energy, novel suspensions, which combine energy regeneration and hydraulic interconnection features, have been proposed recently and are of great value to the automotive industry.

### 1.1. Interconnected Suspensions

The “interconnected” idea of suspension was initially proposed by Hawley in 1927 as “oil pipe interconnected shock absorber” [14]. Since then, various layouts of interconnected suspension and research on the corresponding modeling have been carried out. At present, the main layouts of hydraulic interconnected suspensions are anti-roll [15], anti-pitch [16], anti-bounce and diagonal layout [17], etc. Among them, the most widely studied one is the anti-roll interconnected suspension, whose modeling is highlighted here.

Zhang et al. [18] proposed a frequency domain method to study the hydraulic interconnected suspension system, derived the coupled frequency-related equations and obtained the free vibration solution and frequency response function of a half-vehicle system. Wang et al. [19] did further research. He applied this method to a 7-degree-of-freedom model and studied the effects of several parameters on the roll, pitch and bounce modes of the vehicle. The results were verified by vehicle drop tests. Wang et al. [20] indicated that the HIS system can improve the vehicle stability in both roll and lateral aspects. Ding et al. [16] extended the application of the HIS system to tri-axle trucks, and the modal analysis results indicated that the HIS system can reduce the pitching motion of the sprung mass while maintaining smoothness.

Time-domain analyses on interconnected suspensions have also been conducted. Zhang et al. [21] proposed a new damper for hydraulically interconnected suspension. The AMESim model of hydraulic interconnected suspension was established and simulated. The results showed that the setting time and the overshoot were, respectively, reduced by 42.2 and 14.7%, and the largest roll angle and steady roll were, respectively, decreased by 9.9 and 5.9°. Wang et al. [22] derived the vehicle dynamics model of the hydraulically interconnected inertial device-spring-damper suspension (HIISDS). Two road excitations were used to verify the effectiveness of the suspension. Cao et al. [23] developed a generalized 14 degree-of-freedom nonlinear vehicle model to evaluate the vehicle dynamics of the interconnected suspension. The results showed that the coupled hydro-pneumatic suspension had considerable potential in enhancing the ride comfort and anti-roll/pitch performances.

The existing modeling methods of the mechanical–hydraulic coupled vehicle systems were either in time domain [21,22,23] or in frequency domain [18,19,20]. The frequency-domain modeling research mostly utilized the transfer matrix method to evaluate the impedance matrix of the hydraulic subsystem. With the model in the frequency domain, the modal analysis of the system could be performed and the system’s own characteristics could be analyzed. The corresponding results disclosed a good agreement with experiments [19].

### 1.2. Energy Harvesting Shock Absorbers

Researchers noticed the energy-dissipating nature of shock absorbers and began to study energy harvesting shock absorbers [24,25]. The number of publications per year on energy regenerative shock absorber has been exponentially increased over the last decade [26], including piezo-electric [27] and electromagnetic [28,29] energy harvesting techniques. Among all the energy harvesting shock absorbers, the hydraulic energy harvesting shock absorber was put into the application earlier than other types, attributable to its relatively simple structure [30]. Wu et al. [31] established a mathematical model of the hydraulic energy regenerative shock absorber and conducted a series of bench tests. The results showed that the peak recovery power reached 505.52 W, and the recovery efficiency was 14.5%. Samn et al. [32] designed a hydraulic regenerative shock absorber and demonstrated that it cannot only collect part of the wasted energy in the suspension system, but also improve the vehicle ride comfort and road holding. Fang et al. [33] presented a hydraulic electromagnetic energy-regenerative shock absorber and demonstrated that the damping force varied with the magnitude of the load current. Guo et al. [34,35] demonstrated the method and steps for the size matching and parameter setting of the hydraulic components in the hydraulic electromagnetic energy-harvesting shock absorber (HESA) system. Test results indicated that the proper matching of the parameters can make the HESA system work efficiently and improve the energy conversion efficiency. Peng et al. [36] applied the HESA in a commercial vehicle suspension and indicated that the root mean square energy regenerative power were 41.72 and 339.88 W on Class-B and Class-C roads at speeds of 30–70 km/h.

### 1.3. Energy Harvesting Hydraulic Interconnected Suspension

To combine the merits of the hydraulic interconnected suspension and the energy harvesting shock absorber, various types of the energy harvesting hydraulic interconnected suspension came into individuals’ sight, with the aim to simultaneously enhance the vehicle dynamics performance and harvest energy.

**Figure 1:**
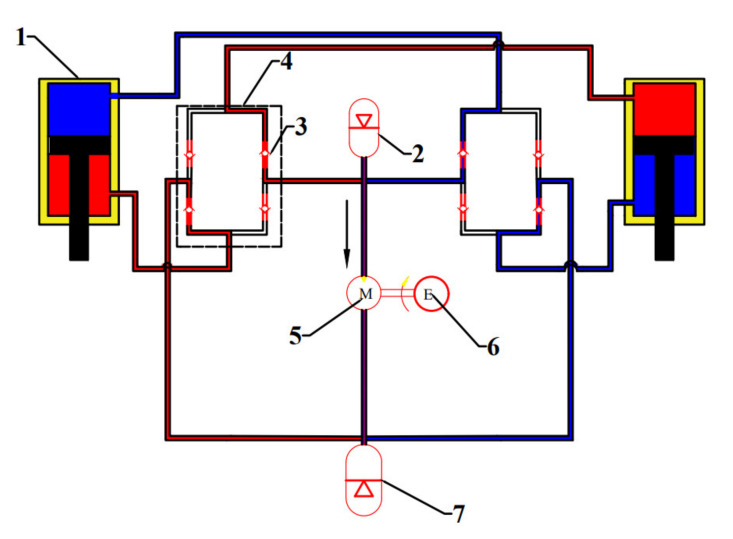
The structure and working principle of the HIIRS. 1 hydraulic cylinder; 2 high-pressure accumulator; 3 check valve; 4 hydraulic rectifier; 5 hydraulic motor; 6 generator; 7 low-pressure accumulator.

Chen et al. [37] integrated the energy harvesting shock absorbers into the hydraulic interconnected suspension system and indicated that the system performed better than the traditional suspension in terms of rolling dynamics and could harvest 421 W energy at 4 Hz and 40 mm (peak) excitation. Guo et al. [38] proposed a hydraulic interconnected suspension system based on hydraulic electromagnetic shock absorbers, which only adopted one set of hydraulic motor-generator system and greatly reduced the cost while improving the energy recovery efficiency. The hydraulic integrated interconnected regenerative suspension (HIIRS) [39], which is studied in this paper, consists of a two-way hydraulic cylinder installed between the wheel and the body, an oil pipe connecting the hydraulic cylinders, a high-pressure accumulator, a low-pressure accumulator, two hydraulic rectifiers and a hydraulic motor-generator unit. Its structure and working principle are shown in Figure 1.

The hydraulic rectifier, composed of four check valves, ensures the one-way flow of fluid to drive the hydraulic motor. There is a high-pressure accumulator at the inlet of the hydraulic motor, which can stabilize the fluid flow through the hydraulic motor. This ensures the hydraulic motor to maintain a stable speed and the generator to generate electricity efficiently. The low-pressure accumulator at the outlet can compensate for the variation of the fluid volume in the HIIRS system. The high-pressure fluid flows through the port of the high-pressure accumulator, and thus, when the high-pressure accumulator is working, it provides an extra rigidity to the suspension.

Recent studies on HIIRS mainly focused on design, modeling and experiments. Their results proved that the energy regenerative suspension had certain energy harvesting capability while ensuring comfort. However, the existing research did not model the HIIRS in the frequency domain. The frequency domain modeling method can easily allow for obtaining the evaluation index of vehicle ride comfort and the energy harvesting power at different road surfaces and vehicle speeds. Compared with the time domain analysis method, the frequency domain analysis method has several distinct advantages when applied to HIIRS. (1) The solution is simpler and more convenient; (2) the natural frequency can be calculated, which could guide the future design of the HIIRS; (3) the frequency domain analysis method can demonstrate the responses of the HIIRS under various excitations in an expedient way. In this paper, a half car model coupled with an HIIRS system is developed in the frequency domain. Based on the model, the vibration isolation characteristics and energy harvesting power of HIIRS are studied.

The rest of the paper is organized as follows. Section 2 develops the model of the HIIRS-equipped half vehicle in the frequency domain based on the block modeling and hydraulic impedance method. Free vibration analysis and forced vibration analysis are performed in Section 3. Energy harvesting power is calculated and estimated in Section 4. Finally, Section 5 concludes this paper.

## 2. Modeling

In this chapter, the idea of modular modeling is adopted. As the HIIRS is introduced into the vehicle, the whole system is divided into two parts: the mechanical system and the hydraulic system. The two parts and their boundary coupling conditions are discussed separately, and finally, the coupled dynamic equation is obtained.

### 2.1. Mechanical System

**Figure 2:**
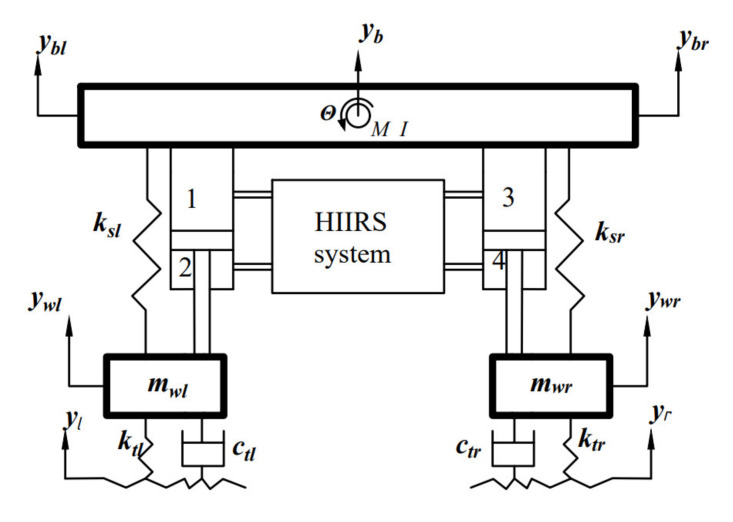
Schematic of a half-car with an HIIRS system.

Considering the simplicity of modeling, while still accounting for fluid interconnections between the wheel stations and the lumped mass, a four-DOF half-car model, as shown in Figure 2, is used in this investigation.

In this section, the mechanical system is what we concerned about, hence the force of the hydraulic system is regarded as an external force. According to Newton’s second law, the kinematics equation of the half-car model is written by

(1) $$M\ddot{y}+C\dot{y}+Ky=f\left(t\right)$$

where y is the displacement vector, y = [$y_{wl}$,$y_{wr}$,$y_{b}$,θ]; $f\left(t\right)$ is the resultant force applied to the vehicle, which can be written as $f\left(t\right)=D_{1}Ap\left(t\right)+f_{x}\left(t\right).$ In the equation of $f\left(t\right)$, $D_{1}Ap\left(t\right)$ is the hydraulic cylinder force, and $f_{x}$ is other external forces vector. We define the pressure vector as $p\left(t\right)={\left[P_{1}\;P_{2}\;P_{3}\;P_{4}\right]}^{T}$ and the area matrix as *A = diag*$\left(A_{1},A_{2},A_{3},A_{4}\right)$. $D_{1}$ is a linear transformation matrix $D_{1}=\left[\begin{array}{cccc} -1 & 1 & 0 & 0\\ 0 & 0 & -1 & 1\\ 1 & -1 & 1 & -1\\ -b_{l} & b_{l} & b_{r} & -b_{r} \end{array}\right]$. $A_{i}$ is the cross-sectional area of the hydraulic cylinder chamber in Figure 1, $b_{l}$ and $b_{r}$ are, respectively, the distances from the plane of gravity center of the vehicle to the left and right suspension.

Equation (1) can now be rewritten as

(2) $$M\ddot{y}+C\dot{y}+Ky=D_{1}Ap\left(t\right)+f_{x}\left(t\right)$$

where $M=\left[\begin{array}{cccc} m_{l} & 0 & 0 & 0\\ 0 & m_{r} & 0 & 0\\ 0 & 0 & M & 0\\ 0 & 0 & 0 & I \end{array}\right]$, $C=\left[\begin{array}{cccc} c_{tl} & 0 & 0 & 0\\ 0 & c_{tr} & 0 & 0\\ 0 & 0 & 0 & 0\\ 0 & 0 & 0 & 0 \end{array}\right]$,

$K=\left[\begin{array}{cccc} k_{sl}+k_{tl} & 0 & -k_{sl} & b_{l}k_{sl}\\ 0 & k_{sr}+k_{sl} & -k_{sr} & -b_{r}k_{sr}\\ -k_{sl} & -k_{sr} & k_{sl}+k_{sr} & -b_{l}k_{sl}+b_{r}k_{sr}\\ b_{l}k_{sl} & -b_{r}k_{sr} & -b_{l}k_{sl}+b_{r}k_{sr} & b_{l}^{2}k_{sl}+b_{r}^{2}k_{sr} \end{array}\right]$.

### 2.2. Mechanical–Fluid System Boundary Conditions

In a double-acting hydraulic actuator cylinder, any piston movement will cause liquid to flow into or out of the chamber of the cylinder. Assuming the pressure difference between the upper and lower chambers produces linear leakage at the piston [40], then the linear leakage of the left and right actuators, $q_{l}$ and $q_{r}$, are given by

(3) $$\left\{\begin{array}{c} q_{l}=\frac{p_{v1}-p_{v2}}{R_{l}}\\ q_{r}=\frac{p_{v3}-p_{v4}}{R_{r}} \end{array}\right.$$

where $R_{l}$ and $R_{r}$ are linearized loss coefficients.

**Figure 3:**
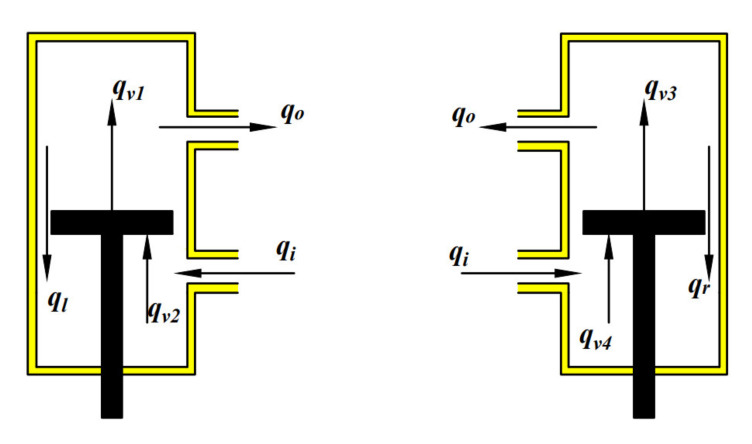
The mechanical–fluid system boundary condition.

The mechanical–fluid boundary condition, shown in Figure 3, are given by

(4) $$\left\{\begin{array}{c} q_{v1}=q_{A1}-q_{l}\\ q_{v2}=q_{A2}-q_{l}\\ q_{v3}=q_{A3}-q_{r}\\ q_{v4}=q_{A4}-q_{r} \end{array}\right.$$

where $q_{Ai}$(*i* = 1 2 3 4) is the fluid volume caused by piston motion, which equals the product of the relative speed of the piston v and the piston area *A*.

For a small roll angle, the relative speed of left and right pistons is

(5) $$\left\{\begin{array}{c} v_{l}=\dot{y_{wl}-}\dot{y_{b}}+b_{l}\dot{\theta }\\ v_{r}=\dot{y_{wr}-}\dot{y_{b}}-b_{r}\dot{\theta } \end{array}\right.$$

Combining Equations (3)–(5), there are

(6) $$q\left(t\right)=AD_{2}\dot{y}+Rp\left(t\right)$$

where $p\left(t\right)$ is the flow vector,$p\left(t\right)={\left[P_{1}\;P_{2}\;P_{3}\;P_{4}\right]}^{T}$.The matrix $D_{2}$ and R are defined as $D_{2}=\left[\begin{array}{cccc} 1 & 0 & -1 & b_{l}\\ 1 & 0 & -1 & b_{l}\\ 0 & 1 & -1 & -b_{r}\\ 0 & 1 & -1 & -b_{r} \end{array}\right]$, $R=\left[\begin{array}{cccc} -1/R_{l} & 1/R_{l} & 0 & 0\\ -1/R_{l} & 1/R_{l} & 0 & 0\\ 0 & 0 & -1/R_{r} & 1/R_{r}\\ 0 & 0 & -1/R_{r} & 1/R_{r} \end{array}\right]$.

### 2.3. Fluid System

In order to solve Equations (2) and (6), which relate to the mechanical system and mechanical–fluid boundary, the fluid system equation in the form $q=f\left(p\right)$ must be obtained. $f\left(p\right)$ depends on the modelling approach used. For the sake of computational efficiency and analytical advantages, only the linear function between p and q is considered. In particular, the focus of this study is frequency domain modeling. Therefore, the target is to seek the linear relationship between the flow rate *Q(s)* and the pressure $P\left(s\right)$ in the frequency domain.

According to the definition of hydraulic impedance, the relationship between the flow rate and the pressure of a fluid system can be expressed as

(7) $$Q\left(s\right)=Z{\left(s\right)}^{-1}P\left(s\right)$$

where *Z* is the impedance matrix.

Equation (6) describes the fluid system of the HIIRS, while Equation (7) describes the mechanical system of the HIIRS. Next, we must combine the two systems, to solve the HIIRS. By comparing the Laplace form of Equations (6) and (7), we can obtain the relationship between the pressure $P\left(s\right)$ and the exciting displacement $Y\left(s\right)$, as

(8) $$P\left(s\right)=sE\left(s\right)AD_{2}Y\left(s\right)$$

where *E(s)* = ${\left(Z{\left(s\right)}^{-1}-R\right)}^{-1}$. Without considering leakage, $E\left(s\right)=Z\left(s\right)$.

The Laplace transform form of Equation (2) is the system differential equation in the frequency domain

(9) $$Ms^{2}Y\left(s\right)+sCY\left(s\right)+KY\left(s\right)=sD_{1}AE\left(s\right)AD_{2}Y\left(s\right)+F_{x}\left(s\right)$$

(10) $$[s^{2}M+s\bar{C}\left(s\right)+K]Y\left(s\right)=F_{x}\left(s\right)$$

where $\bar{C}\left(s\right)=C-D_{1}AE\left(s\right)AD_{2}$.

Defining the state vector $x={\left[y,\;sy\right]}^{T}$, Equation (10) can be rewritten as

(11) $$sX\left(s\right)=\hat{A}\left(s\right)X\left(s\right)+\hat{B}U\left(s\right)$$

where $\hat{A}\left(s\right)=\left[\begin{array}{cc} 0 & I\\ -M^{-1}K & -M^{-1}\bar{C}\left(s\right) \end{array}\right],\;\hat{B}=\left[\begin{array}{c} 0\\ M^{-1} \end{array}\right]$, $U\left(s\right)$ = $F_{x}\left(S\right)$.

**Figure 4:**
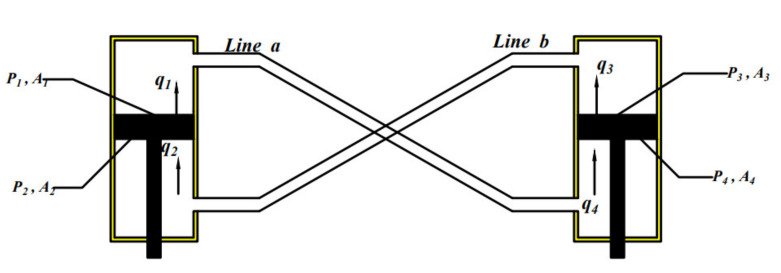
Schematic of a general anti-oppositional half-car HIIRS arrangement.

It should be noted that it is not easy to determine the impedance correlation matrix $\hat{A}\left(s\right)$, which depends on the fluid circuit and the arrangement of the various components. The circuit layout in this study adopts anti-oppositional interconnection, as shown in Figure 4.

Using the original boundary flow definitions, $q_{i}$, the nodal state vectors for the mechanical–fluid interfaces in this arrangement are related by

(12) $$\left[\begin{array}{c} P_{4}\\ Q_{4} \end{array}\right]=\left[\begin{array}{cc} T_{11}^{a} & T_{12}^{a}\\ T_{21}^{a} & T_{22}^{a} \end{array}\right]\left[\begin{array}{c} P_{1}\\ Q_{1} \end{array}\right]\;\left[\begin{array}{c} P_{2}\\ Q_{2} \end{array}\right]=\left[\begin{array}{cc} T_{11}^{b} & T_{12}^{b}\\ T_{21}^{b} & T_{22}^{b} \end{array}\right]\left[\begin{array}{c} P_{3}\\ Q_{3} \end{array}\right]$$

Combining Equation (12) with Equation (7), $Z{\left(s\right)}^{-1}$ can be written as

(13) $$Z{\left(s\right)}^{-1}=\left[\begin{array}{cccc} -\frac{T_{22}^{a}}{T_{21}^{a}} & 0 & 0 & \frac{1}{T_{21}^{a}}\\ 0 & \frac{T_{11}^{b}}{T_{21}^{b}} & T_{12}^{b}-\frac{T_{11}^{b}T_{22}^{b}}{T_{21}^{b}} & 0\\ 0 & \frac{1}{T_{21}^{b}} & \frac{-T_{22}^{b}}{T_{21}^{b}} & 0\\ T_{12}^{a}-\frac{T_{11}^{a}T_{22}^{a}}{T_{21}^{a}} & 0 & 0 & \frac{T_{11}^{a}}{T_{21}^{a}} \end{array}\right]$$

By substituting Equation (13) into Equation (12), it yields the complete system equations.

For Equation (13), the values of the elements in the matrix $T^{a}$ and $T^{b}$ can be obtained by the hydraulic impedance method and the transfer matrix method. In this method, the state vector of the adjacent state node is related to the transfer matrix *T*. If the state vector is defined as fluid pressure *P* and flow rate *Q*, then

(14) $${\left[\begin{array}{c} P\\ Q \end{array}\right]}_{o}=\left[\begin{array}{cc} T_{11} & T_{12}\\ T_{21} & T_{22} \end{array}\right]{\left[\begin{array}{c} P\\ Q \end{array}\right]}_{i}$$

where the subscript o represents the fluid output node, and i represents the fluid input node. As long as the previous output node is regarded as the next input node, according to the fluid flow direction in the circuit, the start node and the end node in the circuit can be connected.

**Figure 5:**
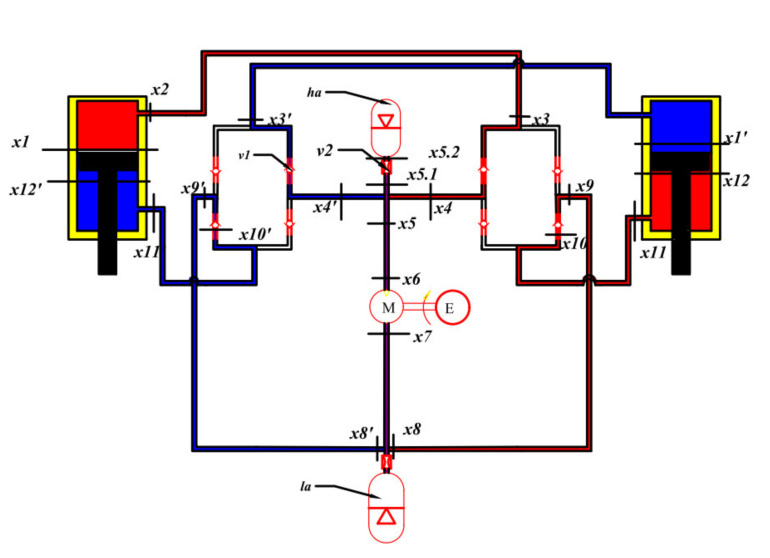
Schematic of a typical half-car with the HIIRS system. v1—check valves; la—low-pressure accumulator; ha—high-pressure accumulator; v2—accumulator valve; x, x′—state nodes.

As the layout of the hydraulic circuit is shown in Figure 5, the transfer matrix $T^{a}$ can be expressed as

(15) $$T^{a}\left(s\right)=T_{X11\to X12}T_{X10\to X11}T_{X9\to X10}T_{X8\to X9}T_{X7\to X8}T_{X6\to X7}T_{X5\to X6}T_{X4\to X5}T_{X3\to X4}T_{X2\to X3}T_{X1\to X2}$$

The transfer matrix of each segment in the circuit depends on the characteristics of the component, which are modeled as follows.

The two-dimensional viscous compressible flow model is applied to model the pipeline, according to [41,42,43]. The basic fluid equations of the model can be summarized into the equation of state, continuity equation and momentum equation:

Equation (16) of state:

(16) $$\frac{dp}{d\rho }=a_{0}^{2}$$

Continuity Equation (17):

(17) $$\frac{\partial p}{\partial t}+\bar{\rho }\left(\frac{\partial v_{x}}{\partial x}+\frac{\partial v_{r}}{\partial r}\frac{v_{r}}{r}\right)=0$$

Momentum Equation (18):

(18) $$\bar{\rho }\frac{\partial v_{x}}{\partial t}=\frac{-\partial p}{\partial x}+\bar{\mu }\left(\frac{\partial^{2}v_{x}}{\partial r^{2}}+\frac{1}{r}\frac{\partial v_{x}}{\partial r}\right)$$

where $v_{x}$ and $v_{r}$ are the axial and radial velocity of fluid; $a_{0}=\sqrt{\bar{\beta }/\bar{\rho }}$ is the fluid sonic velocity; $\bar{\beta }$, $\bar{\mu }$ and $\bar{\rho }$ are the mean value of fluid bulk modulus, viscosity and density. The field transfer matrix of the pipelines is

(19) $$T_{p}=\left[\begin{array}{cc} \cosh\Gamma \left(s\right) & -Z_{C}\left(s\right)\sinh\Gamma \left(s\right)\\ -\frac{\sinh\Gamma \left(s\right)}{Z_{C}\left(s\right)} & \cosh\Gamma \left(s\right) \end{array}\right]$$

where $Z_{C}\left(s\right)=\frac{a\bar{\rho }}{A}{\left(l-\frac{2J_{1}\left(ir\sqrt{s/\bar{v}}\right)}{ir\sqrt{s/\bar{v}}J_{0}\left(ir\sqrt{s/\bar{v}}\right)}\right)}^{-1/2},$ $\Gamma \left(s\right)=\frac{ls}{a}{\left(l-\frac{2J_{1}\left(ir\sqrt{s/\bar{v}}\right)}{ir\sqrt{s/\bar{v}}J_{0}\left(ir\sqrt{s/\bar{v}}\right)}\right)}^{-1/2}$, and *l* is the length of the line element, *A* is the pipeline internal cross-sectional area, *r* is the pipeline internal radius, $\bar{\rho }$ is the mean fluid density, $\bar{v}$ is the mean fluid kinematic viscosity, and $J_{0}$ and $J_{1}$ are Bessel functions of the first kind with orders zero and one, respectively. Considering the compressibility of fluid and pipeline, the propagation velocity of transmission line is

(20) $$a=\sqrt{\frac{a_{0}^{2}}{1+\left(2ra_{0}^{2}\bar{\rho }/t_{p}E\right)}}$$

where $t_{p}$ is the thickness of the pipe wall, and *E* is the Young’s modulus of the pipe material.

The strict model of the flow dynamics through the damping valves usually involves complicated geometrical parameters. The simplified model used here assumes that the damping valve has a negligible fluid volume. The transfer matrix of the damping valves is then expressed as

(21) $$\Omega_{V}=\left[\begin{array}{cc} 1 & -Z_{v}\\ 0 & 1 \end{array}\right]$$

where $Z_{v}=R_{v}$, and $R_{v}$ is the constant linear pressure loss coefficient.

For the model of the accumulator, the following assumptions have been made. The compressibility of the liquid is far lower than that of the gas in the accumulator; the elasticity of the diaphragm is neglected; there is no heat exchange between the gas in the accumulator and the outside world. The axle moves quickly relative to the small amplitude of the car body, resulting in a rapid reduction or expansion in the volume of the accumulator, and satisfies the gas adiabatic balance equation. At this time, the accumulator can be regarded as a linear system [16], and the impedance of the accumulator is

(22) $$Z_{A}=-\frac{\gamma {\bar{P}}^{2}}{sp_{p}v_{p}}$$

The three-way junctions and the accumulator are connected, and hence, the transfer matrix of the three-way junctions and the accumulator are combined here. According to impedance definition and accumulator impedance

(23) $$P_{X_{5.2}}=Q_{X_{5.2}}Z_{A}$$

Set the forward flow direction to be outside the accumulator

(24) $$P_{X5.1}=P_{X5.2}-Z_{V2}Q_{X5.2}\;and\;Q_{X5.2}=Q_{X5.1}$$

Combining Equations (23) and (24), we obtain

(25) $$\frac{P_{X5.1}}{Q_{X5.1}}=Z_{A}-Z_{V2}=Z_{X5.1}$$

Now, applying the fluid continuity equation at the tee-junction yields

(26) $$Q_{X5}=Q_{X4}+\frac{P_{X5.1}}{Z_{X5.1}}$$

Ignoring the pressure difference between the three nodes, the transfer matrix can be written as

(27) $$\Omega_{J}=\left[\begin{array}{cc} 1 & 0\\ \frac{1}{Z_{A}-Z_{V2}} & 1 \end{array}\right]$$

The flow rate at the inlet of the hydraulic motor is defined as $Q_{M}$. One part of $Q_{M}$, which drives the rotation of the hydraulic motor is $\eta_{V}Q_{M}$, where $\eta_{v}$ is the volumetric efficiency of the hydraulic motor. The other part of the flow is the leakage flow $\Delta Q_{M}$ from the high-pressure cavity to the low-pressure cavity. The flow at the outlet of the hydraulic motor is $\eta_{V}Q_{M}$.

When the oil flows through the motor, the relationship between the flow rate $Q_{m}$ of the hydraulic motor and the speed $n_{m}$ (rev/s) of the hydraulic motor is

(28) $$n_{m}=\frac{Q_{M}\eta_{v}}{Q_{m}}$$

Assuming that the equivalent moment of inertia of the motor-generator coupling system is $J_{m}$, the speed of the motor is $n_{m}$, $n_{m}$=$\frac{\omega }{2\pi }$, and ω is the angular velocity. Then, there is a torque balance

(29) $$T_{m2}=T_{e}+J_{m}\dot{\omega }$$

With reference to the principle of hydraulic transmission, the input torque $T_{m1}$ and output torque $T_{m2}$ of the hydraulic motor can be calculated according to the following Equation (30)

(30) $$\left\{\begin{array}{c} T_{m1}=\frac{\Delta P_{m}Q_{m}}{2\pi }\\ T_{m2}=T_{m1}\eta_{m} \end{array}\right.$$

where $\Delta p_{m}$ is the pressure drop, $\eta_{m}$ is the total efficiency of the hydraulic motor, $\eta_{m}=\eta_{v}\eta_{p}$ is the product of the volumetric efficiency and pressure utilization efficiency.

Combining Equations (26)–(30), we obtain

(31) $$\frac{\Delta P_{m}Q_{m}}{2\pi }\eta_{m}=T_{e}+2\pi J_{m}\dot{n_{m}}$$

The electromagnetic induction torque $T_{e}$ of the generator is determined by the armature current I and the torque constant $k_{t}$ of the motor

(32) $$T_{e}=k_{t}I$$

The winding armature current I is related to the design of the energy-regenerative circuit, and the equation can be obtained from Kirchhoff’s voltage law, as

(33) $$U_{emf}=k_{e}\omega$$

Combining Equations (31)–(33) to obtain

(34) $$\Delta p_{m}=\frac{4\pi^{2}k_{e}k_{t}\eta_{v}}{\left(R_{1}+R_{2}\right)Q_{m}^{2}\eta_{m}}Q_{M}+\frac{4J_{m}\pi^{2}\eta_{v}}{Q_{m}^{2}\eta_{m}}\dot{Q_{M}}$$

Laplace transform of the above Equation (34) yields

(35) $$\Delta P_{m}=\frac{4\pi^{2}k_{e}k_{t}\eta_{v}}{\left(R_{e}+R_{\mathrm{in}}\right)Q_{m}^{2}\eta_{m}}Q_{M}+\frac{4J_{m}\pi^{2}\eta_{v}}{Q_{m}^{2}\eta_{m}}sQ_{M}$$

where $R_{e}$ is the external resistance, $R_{in}$ is the circuit resistance, $k_{e}$ is the motor speed constant, $Q_{M}$ is the motor inlet flow, $Q_{m}$ is the motor displacement, $\eta_{v}$ is the volumetric efficiency, and $J_{m}$ is the motor-generator rotational inertia.

Therefore, the impedance matrix of the motor-generator unit is

(36) $$Z_{M}=\frac{\Delta P_{M}}{Q_{M}}=\frac{4\pi^{2}k_{e}k_{t}\eta_{v}}{\left(R_{e}+R_{\mathrm{in}}\right)Q_{m}^{2}\eta_{m}}+\frac{4J_{m}\pi^{2}\eta_{v}}{Q_{m}^{2}\eta_{m}}s.$$

The transfer matrix of the combined model of hydraulic motor and generator can be written as

(37) $${\left[\begin{array}{c} P\\ Q \end{array}\right]}_{out}=\left[\begin{array}{cc} 1 & -Z_{M}\\ 0 & \eta_{v} \end{array}\right]{\left[\begin{array}{c} P\\ Q \end{array}\right]}_{in}$$

Now, the hydraulic impedance and transfer matrix of the pipeline, check valve, accumulator and motor-generator are obtained. Substituting Equations (19), (21), (27) and (36) into Equation (15), $T^{a}$ and $T^{b}$ can be clear. By substituting the relative elements of $T^{a}$ and $T^{b}$ into Equation (13), the matrix $Z^{-1}\left(s\right)$ is obtained with definite elements. As a result, the governing equation of the HIIRS system is determined. Then, Equations (10) and (11) can be applied to solve the vibration problem of the HIIRS-equipped half-vehicle.

## 3. Vibration Analysis

After the above work, the system Equation (11) can be used to analyze the vibration characteristics of the half-car model equipped with HIIRS. The values of each parameter in this study are shown in Table 1.

### 3.1. Free Vibration Analysis

When the external input is zero input, the half-vehicle system vibrates freely, and the corresponding mathematical description is the homogeneous form of Equation (11) as

(38) $$\mathrm{sX}\left(s\right)=\hat{A}\left(s\right)X\left(s\right)$$

The solution of the free vibration of the system can be obtained by solving

(39) $$\det\left(\hat{A}\left(s\right)-\mathrm{sI}\right)=0$$

To solve Equation (38), we must solve the eigenvalue of $\hat{A}\left(s\right)$. Several elements of $\hat{A}\left(s\right)$ are functions of s, unless the frequency is known, $\hat{A}\left(s\right)$ cannot be completely determined, and therefore, the solution of Equation (38) cannot be obtained by conventional methods. The method used here transforms the process of finding the root of the characteristic equation into the process of finding the local optimal solution.

Assuming that the minimum value of the characteristic equation is the objective function, that is, min $J\left(s\right)=\left|\hat{A}\left(s\right)-SI\right|$ is the objective function, the fminsearch function in MATLAB is used to find the local minimum of the objective function J(s). The specific method is mainly divided into two processes. In the first process, the relatively rough root finding is performed. The value of the objective function is calculated after initializing the Laplacian operator. If the local extremum is not found, the value of the Laplacian operator is reinitialized, and the above process is performed again. If the local extremum is found, then the following second process is performed. The second process is a test process, and the Laplacian found in the first process is substituted into the characteristic matrix to obtain a fixed eigen matrix. The eigenvalue of the fixed value matrix is then solved, and the value is compared with the local extremum found in the first process. If the two values are not equal, skip the second process and return to the first process to find the local eigenvalue again. If the two values are the same, the root finding process ends.

With the determined eigenvalues $\lambda_{i}$ and eigenvectors $\alpha_{i}$ from Equation (38), the natural frequency and damping ratio for each mode are given by

(40) $$f_{n_{i}}=\frac{\sqrt{{\left(real\left(\lambda_{i}\right)\right)}^{2}+{\left(imag\left(\lambda_{i}\right)\right)}^{2}}}{2\pi }\;\mathrm{and}\;\xi_{i}=\frac{\left|\left(real\left(\lambda_{i}\right)\right)\right|}{\sqrt{{\left(real\left(\lambda_{i}\right)\right)}^{2}+{\left(imag\left(\lambda_{i}\right)\right)}^{2}}}$$

**Figure 6:**
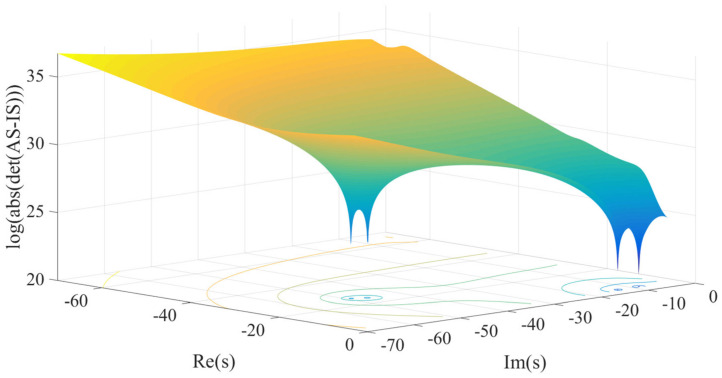
Three-dimensional plot showing the four approximate roots of the characteristic equation of the HIIRS-equipped vehicle.

Based on the analysis of the above complex modal vibration theory, as long as the eigenvalues of the characteristic matrix in Equation (37) and the corresponding eigenvectors are solved, the natural frequency, damping ratio and main vibration mode of the system can be obtained. In order to find the roots conveniently, the three-dimensional graph is obtained, whose horizontal and vertical coordinates are the real and imaginary parts of the Laplacian operator, and the vertical coordinates are the objective function to initially determine the number of roots and the range of the roots according to the relevant parameters of the system. The three-dimensional image of the half-vehicle model obtained is shown in Figure 6.

It is not difficult to see from Figure 6 that the system eigen matrix has four eigenvalues. The system eigenvalues obtained by the local optimization method after initially determining the range of the eigenvalues are shown in Table 2.

The relatively rough roots obtained in the first process need to be tested in the test process. Substituting the eigenvalues in Table 2 into matrix $\hat{A}\left(s\right)$, the system matrix can be completely determined. The traditional methods of finding eigenvalues and eigenvectors can be applied. If the approximate root in process one is equal to the result obtained by the traditional method, it can be considered that the approximate root is the eigenvalue of matrix $\hat{A}\left(s\right)$. The natural frequency, damping ratio and mode shape corresponding to the eigenvalues that passed the inspection are shown in the Table 3.

The results show that the half-vehicle roll model has four main vibration modes. The first-order vibration mode is dominated by the vertical vibration of the vehicle body and the corresponding natural frequency is 1.49 Hz and the damping ratio is 0.40. Usually, this natural frequency of off-road vehicles is 1.3~2 Hz, and the damping ratio is 0.2~0.4 [44]. The second-order mode is dominated by the body roll vibration. The third-order vibration is dominated by wheel vibration, and the two wheels move in the same direction. The fourth-order vibration mode is dominated by wheel vibration, and the wheels on the left and right sides do reverse vibration. At this time, the body does not vibrate vertically, but has a slight roll vibration.

### 3.2. Forced Vibration Analysis

#### 3.2.1. Frequency Response Matrix

When analyzing the vibration of a vehicle model, the general work to be done is to find the vibration transfer function, which can characterize the amplitude and phase of the vehicle model under different frequency excitations. The vibration transfer matrix of the HIIRS-equipped half-car model is solved as follows.

When the road roughness is used as input, Equation (10) can be obtained as

(41) $$[s^{2}M+s\bar{C}\left(s\right)+K]Y\left(s\right)=F_{x}\left(s\right)$$

where $F_{x}\left(s\right)=\vec{F}\left(s\right)\vec{\xi }\left(s\right)$ is the force exerted by the road on the tire, $\vec{\xi }\left(s\right)={\left[\xi_{l},\xi_{r},0,0\right]}^{T}$ is the road excitation. $\vec{F}$ is a 4 × 4 matrix whose elements are zero except for the first two diagonals, which are $\vec{F_{11}}\left(s\right)=k_{tl}+sc_{tl}$ and $\vec{F_{22}}\left(s\right)=k_{tr}+sc_{tr}$.

Equation (41) can be written as

(42) $$B\left(s\right)Y\left(s\right)=\bar{F}\left(s\right)\bar{\xi }\left(s\right)$$

Now, the frequency response matrix of the half-car system can be defined as

(43) $$H_{y}\left(s\right)=\frac{Y\left(s\right)}{\bar{\xi }\left(s\right)}=B^{-1}\left(s\right)\bar{F}\left(s\right)$$

With *s = jω*, the frequency response matrix describes the system displacement response to any excitations. Therefore, as long as the excitation frequency is known, the HIIRS system equation can be completely determined, and the vibration analysis can be carried out just like other linear vehicle models.

#### 3.2.2. The Response to Random Road Excitation

Frequency domain analysis, similar to time domain analysis, is an important method for studying vehicle system vibration. In the frequency domain analysis map, the independent variable is the frequency and the dependent variable is the amplitude of the signal to be analyzed. In Section 2, the model of the mechanical hydraulic coupling system is established. Now, the input spectrum of the road is necessary to obtain the response of the HIIRS system to the excitation of the road.

Road input is the source of the vehicle vibration system. Whether the impact of the vehicle on the road can be obtained, obtaining accurate road information is the key. In the frequency domain, the power spectral density function is generally used to describe the vibration of the random vibration system. The road power spectrum density function is mainly used in vehicle dynamic response, optimal control of suspension, calculation of road load, etc. The vibration response of the vehicle can be evaluated through the road roughness power spectrum and the dynamic characteristics of the vehicle system. If the frequency response of the suspension is waiting to be solved on the basis of the above transfer function, a model of the road input is also needed. Since the car is excited by the unevenness of the road through the tire contacting the ground, it can be known from the random vibration theory that its vibration response is a smooth random vibration. The research object of this paper is a half-car model. The dynamic response characteristics can be obtained by determining the road input spectrum of the left and right wheels.

Suppose that the spatial frequency n represents the road self-power spectral density, $S_{q}\left(n\right)$ and n=1λ holds. When the vehicle is driving on the road at speed *u*, it has

(44) f=un

where *f* is the time frequency. When the vehicle speed does not change, the time-domain frequency bandwidth Δf has the following relationship with the corresponding spatial domain frequency bandwidth Δn

(45) Δf=u·Δn

The power spectral density $S_{q}\left(n\right)$ at the frequency in the spatial domain can be expressed as

(46) $$S_{q}\left(n\right)=\underset{\Delta n\to 0}{\lim}\frac{\sigma_{q\sim \Delta n}^{2}}{\Delta n}$$

where $\sigma_{q\sim \Delta n}^{2}$ is the energy of the road power spectrum in the frequency domain bandwidth Δn. When the vehicle speed is constant, the harmonic components of the road roughness displacement contained in the time band Δf corresponding to the spatial frequency band Δn are the same, so the road power spectral density in the time domain is

(47) $$S_{q}\left(f\right)=\underset{\Delta f\to 0}{\lim}\frac{\sigma_{q\sim \Delta n}^{2}}{\Delta f}$$

By Equations (44)–(47), $S_{q}\left(f\right)$ can be written as

(48) $$S_{q}\left(f\right)=\frac{1}{u}S_{q}\left(n\right)$$

Under normal circumstances, $S_{q}\left(n\right)$ can be directly calculated. The selection of *n* in the equation is related to the speed and frequency of the driving vehicle. If the condition that 5 m/s < *u*< 50 m/s and 0.5 Hz < *f* < 50 Hz are satisfied at the same time, there is $0.01m^{-1}<n<10m^{-1}$.

It can be seen from the experiment that $S_{q}\left(n\right)$ can be written as

(49) $$S_{q}\left(n\right)=cn^{-2w}$$

where w is a coefficient ranging from 1–1.25 and is generally taken as 1. The value of *c* is related to the road surface level, shown in Table 4.

When the frequency index is taken as 2, the road power spectral density $G_{q}\left(f\right)$ satisfies

(50) $$G_{q}\left(f\right)=G_{q}\left(n_{0}\right)n_{0}^{2}\frac{u}{f^{2}}$$

where the reference spatial frequency $n_{0}$ is taken as 0.1. According to Equations (49) and (50), we can obtain

(51) $$S_{q}\left(f\right)=c\frac{u}{f^{2}}$$

Considering the actual coherence of the left and right wheels, the road input spectral density matrix of the half-vehicle model is

(52) $$S=\left[\begin{array}{cc} S_{D} & S_{X}\\ S_{X} & S_{D} \end{array}\right]$$

where $S_{D}$ is the self-power spectral density of the road excitation

(53) $$S_{D}=\frac{1}{u}S_{q}\left(n\right)=\frac{1}{u}cn^{-2w}$$

The road cross power spectral density $S_{X}$ is

(54) $$S_{X}=\frac{1}{u}\left[2c{\left(\frac{\pi L}{n}\right)}^{w}/\Gamma \left(w\right)\right]J_{w}\left(2\pi Ln\right)$$

where *L* is the left and right wheel track, $J_{w}$ is the second-class modified Bessel function of order *w*, and $\Gamma \left(w\right)$ is the gamma function.

When the left and right wheels are excited by the road roughness transfer function, the relationship between the response spectrum and the input spectrum in the frequency domain is as

(55) $$S_{i}\left(f\right)=\left[H_{i1}^{*}\;H_{i2}^{*}\right]S\left[\begin{array}{c} H_{i1}\\ H_{i2} \end{array}\right]$$

where * represents the conjugate complex number, $S_{i}\left(f\right)$ represents the power spectrum of the *i*-th output, $H_{i1}$ and $H_{i2}$, respectively, represent the transfer function of the *i*-th output to the first and second inputs.

**Figure 7:**
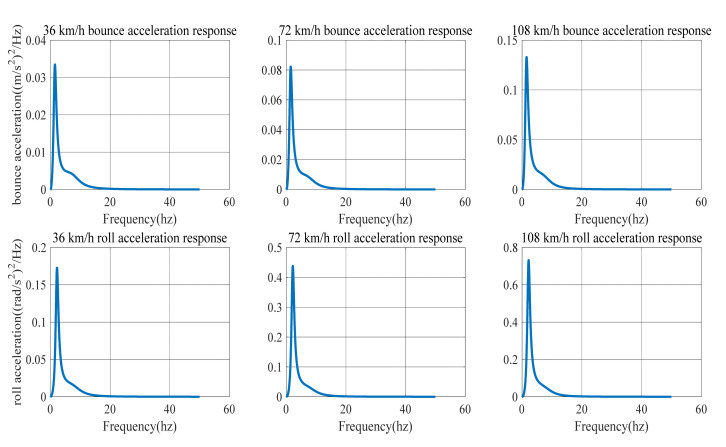
The power spectrum density of the acceleration of an HIIRS-equipped vehicle when driven at 36, 72 and 108 km/h on Class-B road.

When $c=64\times 10^{-8}$ (Class-B road) and the vehicle speed is 36, 72 and 108 Km/h, the power spectrum density of bounce acceleration and the power spectrum density of roll acceleration of the HIIRS system are shown, respectively, in Figure 7.

**Figure 8:**
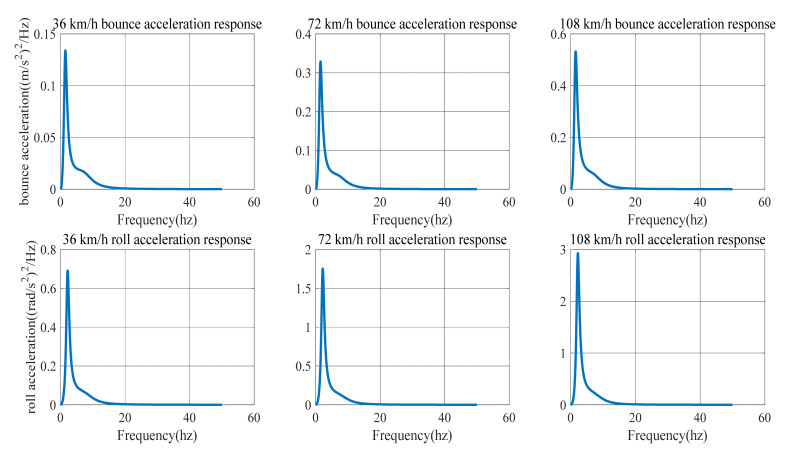
The power spectrum density of the bounce/roll acceleration of an HIIRS-equipped vehicle when driven at 36, 72 and 108 km/h on Class-C road.

When $c=256\times 10^{-8}$ (C-level road) and the vehicle speed is 36, 72 and 108 km/h, the power spectrum density of bounce acceleration and the power spectrum density of roll acceleration of the HIIRS system are shown, respectively, in Figure 8.

Figure 7 and Figure 8 show the power spectrum density of the bounced acceleration and roll acceleration of the vehicle body. The natural frequency and root mean square (RMS) of the acceleration response under Class-B and Class-C roads are in Table 5. It indicates that the natural frequency of bounce is around 1.5 Hz, while the roll frequency is around 2.19 Hz. In addition, an increase in speed on the same road will lead to an increase in the acceleration power spectrum density. At the same speed, an improvement in road conditions will reduce the acceleration power spectrum.

No matter what kind of road surface excitation, the HIIRS system has the same modal law: no matter the speed is low, medium or high, the natural frequency of the roll mode always maintains at around 2.19 Hz, and the natural frequency of the bounce mode varies when the vehicle speed increases. The natural frequency is 1.48 Hz at low speed, 1.50 Hz at medium speed and 1.51 Hz at high speed. This indicates that when the vehicle speed increases, the HIIRS system can provide greater rigidity. In terms of amplitude, the RMS bounce acceleration at medium speed increased by 143.66% compared with that at low speed, while the RMS bounce acceleration at high speed increased by 63.54% compared with that at medium speed. The RMS roll acceleration at medium speed increased by 149.59% compared with that at low speed, while the RMS roll acceleration at high speed increased by 67.63% compared with that at medium speed. From this point of view, it can be seen that as the vehicle speed increases, the RMS acceleration also increases, but the amount of increase at high speed is less than that at low speed. To a certain extent, it indicates that the anti-bounce and anti-roll capabilities of the HIIRS system are more greatly enhanced at high speeds.

**Figure 9:**
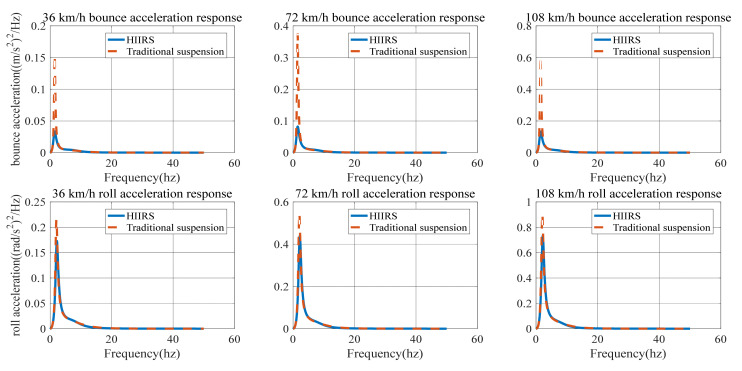
Comparison of bounce/roll acceleration between the HIIRS-equipped vehicle and the traditional vehicle when driven at 36, 72 and 108 km/h on Class-B road.

Figure 9 indicates that the acceleration power spectral density curve of the HIIRS system has the same trend with the traditional suspension. The difference is that the peak value of the HIIRS system is much lower than that of the traditional suspension. Additionally, the improvement is more noticeable in the vertical acceleration than the roll acceleration.

Figure 9 compared the power spectrum density of bounce/roll acceleration between the HIIRS-equipped vehicle and the traditional vehicle, when the vehicles are driven on a Class-B road ($c=64\times 10^{-8})$. In the traditional vehicle, the stiffness and damping are, respectively, 80000 and 4400 Ns/m. The corresponding results are also summarized in Table 6.

From the natural frequency aspect, the roll natural frequency of the HIIRS system and traditional suspension system are maintained at 2.19 and 2.04 Hz, respectively, while the bounce natural frequency of HIIRS ranges from 1.48 to 1.51 HZ and that of traditional suspension is about 1.48 HZ. This indicates that when the road surface and speed are the same, the HIIRS system can provide greater stiffness, especially when the vehicle rolls, since the roll natural frequency of the HIIRS system is higher than that of the traditional suspension.

From the amplitude aspect, Figure 9 shows that the response of the HIIRS is lower than the traditional suspension in the whole frequency range, and the HIIRS can greatly reduce the peak value of the responses. Table 6 shows the RMS of bounce and roll acceleration of the HIIRS system can, respectively, reach 64.91 and 12.38% lower than the traditional suspension when the vehicle is driven at 72 km/h on Class-B road. That is, the HIIRS system provides superior ride comfort and handling stability to traditional suspension systems.

## 4. Energy Harvesting Power of the HIIRS

Section 3 demonstrated the vibration characteristics of the HIIRS, and this section focuses on the energy harvesting characteristics.

In the energy harvesting circuit, the relationship between the various physical quantities can be written as

(56) $$\left\{\begin{array}{c} P=I^{2}R_{e}\\ I=\frac{U_{emf}}{R_{e}+R_{in}}\\ U_{emf}=k_{e}\omega \\ \omega =2\pi \frac{Q_{M}}{Q_{m}}\eta_{v} \end{array}\right.$$

where *P* is the energy harvesting power, $R_{e}$ is the external resistance, *I* is the current, $U_{emf}$ is the induced electromotive force, $R_{in}$ is the circuit resistance, $k_{e}$ is the speed constant of the generator, $Q_{M}$ is the motor inlet flow, $Q_{m}$ is the motor displacement, $\eta_{v}$ is the volumetric efficiency.

In Equation (56), only $Q_{M}$ is the unknown quantity, and thus, the goal is to solve $Q_{M}$.

According to Figure 4 and Equation (15), the state quantity (pressure, flow) of the motor inlet is

(57) $$T_{X6}=T_{X5\to X6}T_{X4\to X5}T_{X3\to X4}T_{X2\cdot X3}T_{X1\cdot X2}T_{X1}$$

State quantity at node 1 is $T_{X1}=\left[\begin{array}{c} P_{1}\\ Q_{1} \end{array}\right]$, $P_{1}$ and $Q_{1}$ can be calculated according to Equations (7) and (8).

According to Equation (56), the transfer function $HX_{61}$ between the motor inlet flow rate and $T_{X1}$ can be known. Equations (7) and (8) provide the transfer function $HX_{1Y}$ between $T_{X1}$ and the displacement vector Y. Therefore, the transfer function between the motor inlet flow and the displacement vector Y in Equation (1) is

(58) $$H_{Q_{M}Y}=HX_{61}\cdot HX_{1Y}$$

Then, the power spectral density of the flow rate at the motor inlet, $G_{Q_{M}},$ can be calculated as

(59) $$G_{Q_{M}}=H_{Q_{M}Y}^{2}G_{Y}$$

where $G_{Y}$ is the power spectral density of Y.

The power spectral density has the following relationship with the amplitude

(60) $$G_{Q_{M}}=A_{Q_{M}}^{2}/f_{s}$$

where $A_{Q_{M}}$ represents the amplitude of $A_{Q_{M}}$, and $f_{s}$ represents the frequency bandwidth.

In Section 3, we obtained the power spectral density of bounce and roll acceleration under random road input and the frequency response matrix of the displacement vector Y. If the method of solving acceleration power spectral density is extended to displacement, with the known power spectral density of the road displacement vector *Y*, the power spectral density of the flow rate at the motor inlet under various random road input can be obtained. Then, the corresponding amplitude can be solved with Equation (60), and the time domain flow rate at the motor inlet can be solved by performing inverse Fourier transform. The time domain flow rate is then substituted into Equation (56), and the energy harvesting power can be determined.

**Figure 10:**
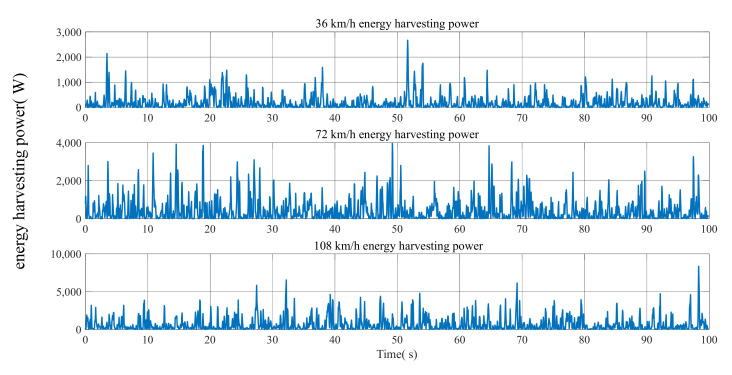
The energy harvesting power of the HIIRS-equipped half-vehicle when driven at various speeds on Class-C road.

The energy regenerative power of a half-car with HIIRS system on a Class-C road at the speed of 36, 72 and 108 km/h is shown in Figure 10. The average value of energy regenerative power at this road surface excitation is shown in Table 7. It shows that the energy harvesting power can reach 655.90 W for an off-road vehicle when it is driven on a Class-C road at 108 km/h.

## 5. Conclusions

This paper studied the vibration isolation and energy harvesting characteristics of a novel hydraulic integrated interconnected regenerative suspension (HIIRS). The model in the frequency domain was established. Both free and forced vibration analysis were carried out and compared with a traditional suspension. The comparison showed that the RMS bounce and roll acceleration of the HIIRS system was, respectively, 64.91 and 12.38% lower than the traditional suspension when the vehicle was driven at 72 km/h on a Class-B road. With the frequency domain model of the HIIRS, an approach for calculating the energy harvesting power was also presented. The calculated energy harvesting power was 186.93, 416.40 and 656.90 W, when the vehicle speed was 36, 72 and 108 km/h. In summary, the HIIRS system can significantly enhance the vehicle ride comfort and handling stability while harvesting vibration energy to achieve an energy-saving purpose.

## Figures and Tables

**Table 1:** Nomenclature.

Symbol	Values	Units	Description
*M*	1400	kg	Sprung mass
*I*	625	${\mathrm{kgm}}^{2}$	Sprung mass moment of inertia about roll axis
$m_{wl},\;m_{wr}$	60	kg	Unsprung mass
$b_{l},\;b_{r}$	0.9	m	Distance from gravity center to suspension strut
$k_{sl},\;k_{sr}$	30	${\mathrm{kNm}}^{-1}$	Mechanical suspension spring stiffness
$k_{tl},\;k_{tr}$	300	${\mathrm{kNm}}^{-1}$	Tire spring stiffness
ρ	870	${\mathrm{kgm}}^{-3}$	Density
μ	0.05	${\mathrm{Nsm}}^{-1}$	Viscosity
$\beta_{oil}$	1400	MPa	Bulk modulus
l	1	m	Length of pipe
$d_{p}$	$16\times 10^{-3}$	m	Pipeline diameter
$V_{ph}$	$4\times 10^{-4}$	$m^{3}$	Pre-charge gas volume of ha
$P_{ph}$	0.8	MPa	Pre-charge pressure of ha
$V_{pl}$	$4\times 10^{-4}$	$m^{3}$	Pre-charge gas volume of la
$P_{pl}$	0.1	MPa	Pre-charge pressure of la
$A_{i}$ $,\;A_{j}$	$2,\;1.6\;(\times 10^{-3}$)	$m^{2}$	Upper and lower piston areas (*i* = 1, 3; *j* = 2, 4)
$s_{j}$	0.15	m	Stroke length
$R_{v1}$	$5\times 10^{8}$	${\mathrm{kgs}}^{-1}m^{-4}$	Linear loss coefficient for check valve
$R_{v2}$	$3\times 10^{8}$	${\mathrm{kgs}}^{-1}m^{-4}$	Linear loss coefficient for accumulator valves
$Q_{m}$	10	cc/rev	Hydraulic motor displacement
$J_{m}$	0.0005	${\mathrm{kgm}}^{2}$	Motor-generator rotational inertia
$k_{t}$	0.25	NM/A	Torque constant
$k_{e}$	0.25	$V/\left(\mathrm{rad}/s\right)$	Speed constant
$R_{e}$	10	Ω	Load Resistance
$R_{in}$	0.6	Ω	Motor internal resistance

**Table 2:** The approximate eigenvalue solutions to the system matrix of the HIIRS-equipped vehicle.

Eigenvalues	First	Second	Third	Fourth
Real part	−3.7850	−3.6440	−24.4428	−25.7550
Imaginary part	−8.5587	−13.1938	−46.8497	−49.1905

**Table 3:** Vibration modes of the HIIRS-equipped vehicle.

Mode	Bounce	Roll	Wheel Hop 1 (Synchronous)	Wheel Hop 2 (Oppositional)
Frequency (Hz) and damping ratio	$f_{n}=1.49$ $\xi_{n}=$0.40	$f_{n}=2.18$ $\xi_{n}=$0.27	$f_{n}=8.41,$ $\xi_{n}=$0.46	$f_{n}=8.84$ $\xi_{n}=$0.46
State variable (s)	−3.79±8.56i	−3.6±13.19i	−24.44±46.85i	−25.76±49.19i
$\mathrm{Left}\;\mathrm{wheel}\;(y_{wl}$)	0.21–0.23i	−0.29 + 0.18i	1	1
$\mathrm{Right}\;\mathrm{wheel}\;(y_{wr}$)	0.21–0.23i	0.29–0.18i	1	−1
$\mathrm{Centre}\;\mathrm{of}\;\mathrm{gravity}\;(y_{v}$)	1	0	−0.02 + 0.08i	0
Roll angle (*θ*)	0	1	0	0.06–0.14i

**Table 4:** The value of c for various road surface level.

Type of Pavement	The Value Range of c
Class A	$8\times 10^{-8}\sim 32\times 10^{-8}$
Class B	$32\times 10^{-8}\sim 128\times 10^{-8}$
Class C	$128\times 10^{-8}\sim 512\times 10^{-8}$

**Table 5:** The natural frequency and RMS acceleration response of the HIIRS-equipped vehicle.

Working Conditions		Class-B Road			Class-C Road		
		36 km/h	72 km/h	108 km/h	36 km/h	72 km/h	108 km/h
Natural frequency	Bounce (Hz)	1.48	1.50	1.51	1.48	1.50	1.51
	Roll (Hz)	2.19	2.19	2.19	2.19	2.19	2.19
RMS of acceleration	$\mathrm{Bounce}\;(m/s^{2})$	0.0049	0.0120	0.0196	0.0109	0.0480	0.0393
	Roll ($rad/s^{2}$)	0.0246	0.0614	0.1029	0.0985	0.2457	0.2059

**Table 6:** The comparison of the natural frequency and RMS bounce/roll acceleration between the HIIRS system and traditional suspension system.

Suspension and Speed		Natural Frequency of Bounce (Hz)	Natural Frequency of Roll (Hz)	$\mathrm{RMS}\;\mathrm{Bounce}\;\mathrm{Acceleration}\;(m/s^{2})$	$\mathrm{RMS}\;\mathrm{Roll}\;\mathrm{Acceleration}\;(rad/s^{2})$
HIIRS	36 km/h	1.48	2.19	0.0049	0.0246
	72 km/h	1.50	2.19	0.0120	0.0614
	108 km/h	1.51	2.19	0.0196	0.1029
Traditional suspension	36 km/h	1.48	2.04	0.0139	0.0276
	72 km/h	1.48	2.04	0.0342	0.0690
	108 km/h	1.48	2.04	0.0554	0.1150

**Table 7:** Energy harvesting power.

Vehicle Speed	36 km/h	72 km/h	108 km/h
Energy harvesting power (W)	186.93	417.39	655.90
